# The Epidemiology of Stevens-Johnson Syndrome and Toxic Epidermal Necrolysis in China

**DOI:** 10.1155/2018/4320195

**Published:** 2018-02-11

**Authors:** Shang-Chen Yang, Sindy Hu, Sheng-Zheng Zhang, Jin-wen Huang, Jing Zhang, Chao Ji, Bo Cheng

**Affiliations:** ^1^Department of Dermatology, Xiamen Chang Gung Hospital, Xiamen, Fujian, China; ^2^Department of Dermatology, Chang Gung Memorial Hospital, Chang Gung University, Taoyuan, Taiwan; ^3^Department of Dermatology, The First Affiliated Hospital of Fujian Medical University, Fuzhou, Fujian, China

## Abstract

Stevens-Johnson syndrome and toxic epidermal necrolysis (SJS/TEN) are life-threatening disease. However, there are only few epidemiologic studies of SJS/TEN from China. To analyze the clinical characteristics, causality, and outcome of treatment for SJS/TEN in China, we reviewed case reports of patients with SJS/TEN from the China National Knowledge Infrastructure (CNKI) and Wanfang database from 2006 to 2016 and patients with SJS/TEN who were admitted to the First Affiliated Hospital of Fujian Medical University during the same period. There were 166 patients enrolled, including 70 SJS, 2 SJS/TEN overlap, and 94 TEN. The most common offending drugs were antibiotics (29.5%) and anticonvulsants (24.1%). Carbamazepine, allopurinol, and penicillins were the most common single offending drugs (17.5%, 9.6%, and 7.2%). Chinese patent medicines accounted for 5.4%. There were 76 (45.8%) patients receiving systemic steroid and intravenous immunoglobulin (IVIG) in combination therapy, especially for TEN (80.3%), and others were treated with systemic steroids alone. Mortality rate of combination treatment comparing with steroid alone in TEN patients had no statistical significance. In conclusion, carbamazepine and allopurinol were the leading causative drugs for SJS/TEN in China. Combination of IVIG and steroids is a common treatment for TEN, but its efficacy in improving mortality needs further investigation.

## 1. Introduction

Stevens-Johnson syndrome/toxic epidermal necrolysis (SJS/TEN) is a well-known severe cutaneous adverse reaction (SCAR) belonged to type IV hypersensitivity, mediated by immunological effect [1]. This hypersensitivity reaction is recognized as a dysregulation of cellular immunity [2], caused by a release of various cytotoxic signals including granulysin [3], perforin/granzyme B, and Fas/Fas ligand [4] which were activated by cytotoxic T lymphocytes and natural killer cells. SJS/TEN refers to a spectrum with widespread epidermal detachment and mucocutaneous involvement [5]. Different total body surface areas (TBSA) of detached or detachable skin lesions as <10%, 10–30%, and >30% are representing Stevens-Johnson syndrome (SJS), SJS/TEN overlap (SJS-TEN), and toxic epidermal necrolysis (TEN) [6]. SCORTEN disease severity scoring system is widely used in assessing the mortality of SJS/TEN [7]. The mortality rates of SJS, SJS-TEN, and TEN were 5–10%, 30%, and 50%, respectively [2, 5]. Recently, IL-15 has been found to be useful in predicting severity and monitoring prognosis [2]. A global population-based study had previously reported that the incidence of SJS and TEN is estimated 1.0 to 6.0 per million and 0.4 to 1.2 per million, respectively [8]. However, Frey et al. [9] estimated that Asian patients were at a 2-fold risk of SJS/TEN when compared with Caucasian patients in their recent study. There are few English literatures related to SJS/TEN studies from China so far. In this study, we analyzed case reports of SJS/TEN from Chinese literatures and cases from a tertiary referral medical center from the past 10 years. The clinical characteristics, common drug causality, and outcome of treatments were analyzed.

## 2. Methods

We reviewed cases of SJS/TEN from the China National Knowledge Infrastructure (CNKI) and Wanfang Data [10–37] from January 2006 to December 2016. CKNI and Wanfang Data were well-known large comprehensive network full-text databases in China, established, respectively, since 1999 and 2000. Data from online database were searched by the key word of Stevens-Johnson syndrome and toxic epidermal necrolysis. All cases from databases were published in Chinese journals. We only enrolled cases which had detailed description of skin lesions, photographs, or histopathologic findings.

In addition, we also analyzed admission database from the First Affiliated Hospital of Fujian Medical University (FJMU) during 2006 to 2016. This hospital is the major tertiary referral medical center in Fujian Province and had total 4006 dermatology inpatients during this period. Data from admission database were searched by the diagnosis of Stevens-Johnson syndromes and toxic epidermal necrolysis. One patient from the FJMU has been published as a case report in Chinese literature.

All cases of SJS/TEN enrolled for this analysis from the CNKI, Wanfang Data, and FJMU fulfilled with RegiSCAR (European Registry of Severe Cutaneous Adverse Reactions) criteria of probable to definite cases. They were carefully assessed by at least two dermatologists and further validated by the Taiwan-SCAR consortium [38–40]. All cases met the criteria of SJS/TEN from databases, and the hospital had been double checked by sex, age, and causality to exclude overlapping. The drug causalities of enrolled cases were assessed by the ALDEN algorithm, only with probable or definite (ALDEN score ≥ 4), and were included as drug-induced SJS/TEN.

All cases in this study were Han Chinese. We analyzed the detailed information collected from reviewed literatures or medical records, including patient demographics (sex and age), offending drugs, underlying medical diseases, treatments, and outcomes. We also further compared the causality of SJS/TEN in China and Southeast Asia [41].

Statistical analyses were performed using SPSS for Windows version 21.0 (IBM, Armonk, NY). Fisher's exact tests were used for analysis. Odds ratio (OR) and 95% confidence interval (CI) were also calculated. *P* < 0.05 (two-tailed) was considered to be statistically significant.

## 3. Results

**Figure 1:**
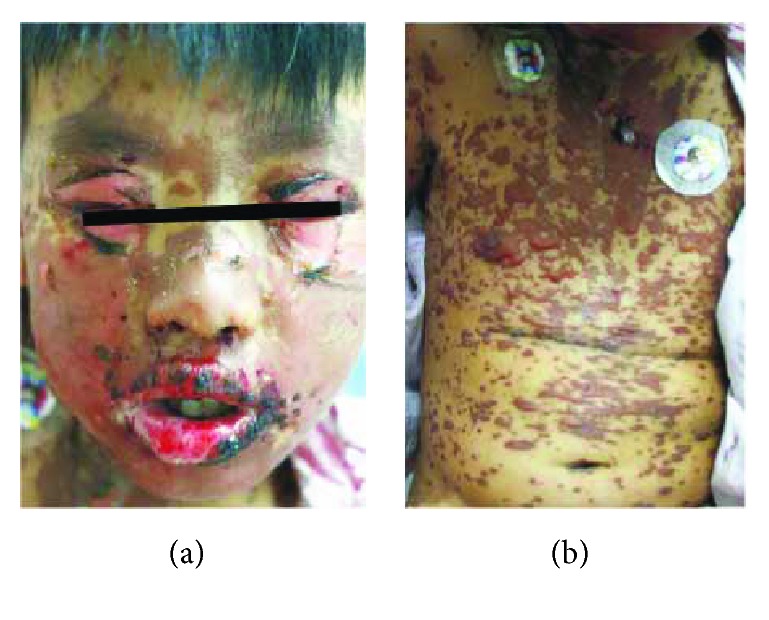
Typical cases of SJS from Chinese literature [20]. (a) Detachment of the eyelids, erosions and crusts of lips, and brownish macules on face and neck with scattered skin detachment. (b) Brownish macules with blisters and detachment on the trunk.

**Figure 2:**
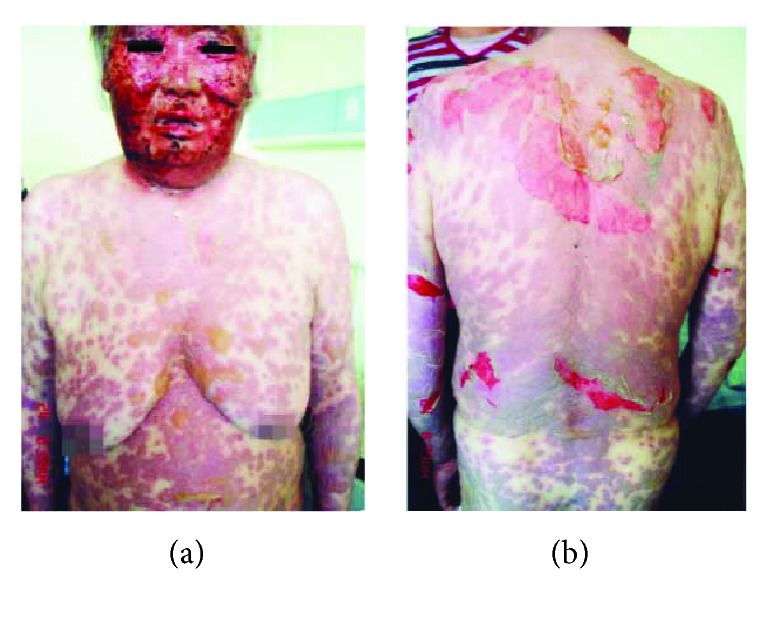
Typical cases of TEN from Chinese literature [12]. (a) Widespread reddish to purplish macules and bullae on the trunk and upper limbs, with erosions on swollen face. (b) Macules and large skin detachment on the lateral trunk and upper limbs.

There were total 230 SJS/TEN cases collected from reported Chinese literatures and admission database from the First Affiliated Hospital of FJMU between 2006 and 2016. Totally, 166 met the criteria of probable to definite cases of SJS/TEN, including 94 cases from literatures and 72 cases from the hospital (incidence rate of hospital population was 1.8%). Among them, there were 70 (42.2%) as SJS, 2 (1.2%) as SJS-TEN, and 94 (56.6%) as TEN. Typical cases of SJS and TEN from Chinese literature were shown in Figures 1 and 2.

### 3.1. Demographic Data, Treatment, and Prognosis of Patients with SJS/TEN

The demographic and characteristics are summarized in Table 1. The age of the onset of SJS/TEN ranged from 1 to 94 years. Mean age of both SJS/TEN is over 40 years, with SJS or SJS-TEN in 43.4 years, and TEN in 43.6 years. There were 46 (63.9%) males and 26 (36.1%) females diagnosed with SJS or SJS-TEN and 54 (57.4%) male and 40 (42.6%) females diagnosed with TEN. There were 4 patients that were found to have HIV positive. All the enrolled patients received systemic corticosteroid, mostly methylprednisolone (67.8 ± 38.4 mg/d). Among these 166 cases, 76 (45.8%) patients had additional intravenous immune globulin (IVIG) (0.5 ± 0.3 g/kg/d), 11 patients received steroid pulse therapy (methylprednisolone 300–500 mg/d), 1 patient had cyclophosphamide, and 1 patient had plasmapheresis.

### 3.2. Causality of SJS/TEN

We categorized the causality into 9 groups in Table 2. The commonest causative drug category for SJS/TEN was antibiotics in 49 (29.5%) patients, and 75.5% of them were diagnosed with TEN. The largest proportion of the identified single offending antibiotics was penicillins (7.2%), followed by cephalosporins (4.2%) and quinolones (3.6%). Many of the patients had concomitant use with multiple antibiotics (4.8%). The second common offending drug category was anticonvulsants (*n* = 40, 24.1%), which the leading cause was carbamazepine (17.5%), followed by lamotrigine (4.2%), oxcarbazepine (1.2%), phenobarbital (0.6%), and phenytoin (0.6%). Three patients among them had undergone HLA genotyping, one carbamazepine-TEN and one oxcarbazepine-SJS carried the risk *HLA-B*^∗^*15 : 02* allele, and the other carbamazepine-SJS carried *HLA-B*^∗^*51 : 01/15 : 11* without *HLA-B*^∗^*15 : 02*. Allopurinol contributed 16 (9.5%) patients, 2 of them had received HLA genotyping and revealed *HLA-B*^∗^*58 : 01* positive.

Chinese patent medicines accounted for 9 (5.4%) cases, mostly were compound preparations. Three were for cold or clearing heat, including cough granule (containing loquat, opium poppy husk, stemona, mulberry bark, swallowwort rhizome, etc.), bupleurum granule (containing bupleurum, *Pinellia ternata* with ginger, radix scutellariae, *Codonopsis pilosula*, etc.), and extract of *Andrographis paniculata*. Others were sleeping capsule (containing lilium, *Acanthopanax senticosus*, caulis polygoni multiflori, Albizia julibrissin durazz, mother-of-pearl, etc.), Gutong capsule (containing ginseng, resina draconis, scorpion, bungarus minimus, etc.), and Honghua tablet (containing *Emilia sonchifolia*, *Hedyotis diffusa*, caulis spatholobi, etc.), and the last three were unspecified.

There were 8 (4.8%) cases caused by nonsteroidal anti-inflammatory drugs (NSAIDs), including diclofenac, ibuprofen, analgin, and some compound which contained paracetamol, caffeine, and aspirin, or aminopyrine, or phenacetin. Ten (6.0%) patients have taken multiple drugs concomitantly for treating common cold, including different combinations of antibiotics, anticonvulsant, NSAIDs, and Chinese patent drugs. There were 3 (1.8%) patients caused by industrial chemicals, which were acetochlor, naphthalenedisulfonic acid dimethyl ester, and trichloroethylene. Finally, 12 (7.2%) patients were caused by other drugs, including methazolamide (*n* = 8), dobesilate (*n* = 1), antifungals (*n* = 1), antidepressant (*n* = 1), and antituberculosis drugs (*n* = 1). One patient using methazolamide had HLA genotyping and was found to be *HLA-B*^∗^*59 : 01* positive. However, 19 (11.4%) patients had no offending drug identified and no known infections.

The distribution of offending drugs causing SJS/TEN in northern or southern China was similar in which antibiotics (30.4% versus 29.2%) and anticonvulsants (28.3% versus 22.5%) were of most causative categories. However, there were more cases of allopurinol-related SJS/TEN in southern China (11.7% versus 4.3%) and more NSAID-related cases in northern China (8.7% versus 3.3%) (Table 3).

We further compared the drug causality of SJS/TEN in China to that in Southeast Asia, and the result was shown in Table 4. The proportion of antibiotics or anticonvulsant-related SJS/TEN of Malaysia (27.8% and 33.3%) and Singapore (28.9% and 29.6%) was similar to that of China (29.5% and 24.1%), Thailand had higher percentage of antibiotic-related SJS/TEN (66.7%), and the Philippines had higher percentage of anticonvulsant-related SJS/TEN (42.9%). Penicillins were the most common causative antibiotics in China in our study (7.2%), which are similar to Singapore (11.9%) and Thailand (31.7%), whereas sulfonamide being the largest group of antibiotics in Malaysia (17.3%) and the Philippines (7.1%). Carbamazepine was the most common causative anticonvulsant in our study (17.5%) and also in other Southeast Asian countries (Table 4). Allopurinol was also one of the leading causes for SJS/TEN in Asian countries (China: 9.6%, Philippines: 21.4%, and Singapore: 20.4%). Interestingly, Chinese patent medicines, or herbal medicines, which are still common traditional therapeutics in Chinese society, caused 7.5% SJS/TEN in Singapore, 5.4% of our study in China, and 3.6% and 2.5% in the Philippines and Malaysia, respectively.

### 3.3. Mortality of SJS/TEN

There were 9 (5.4%) deceased patients (Table 5), 1 was SJS, and 8 were TEN. Patient diagnosed with SJS was a 51-year-old male, with underlying disease of chronic renal failure and diabetes, and had cardiorespiratory arrest before admission. Other 8 patients diagnosed with TEN mostly had cardiovascular disease, diabetes, and nephropathy. Besides a child with age of 3 years, all patients were older than 40 years, ranging from 51 to 94. Among 9 deceased patients, 4 patients received systemic steroids in combination with IVIG, 3 in the early stage and 1 in the late stage, and 5 patients received systemic steroids only.

### 3.4. Treatment with Combination of Steroid and IVIG versus Steroid Alone

There were 90 (54.2%) patients of SJS/TEN who received systemic steroids alone and 76 (45.8%) patients who had IVIG in combination with systemic steroids. Combination treatment was more commonly used in TEN patients than in SJS patients (64.9% versus 20.8%) (Odds ratio: 7.024; *P* < 0.001) (Table 1). In 76 patients who received systemic steroids with IVIG in combination, 61 (80.3%) of them were TEN, and the mortality rate of TEN cases receiving combination treatment was 6.6% (4/61). In 90 patients who received systemic steroids alone, 33 patients (36.7%) were TEN, and 12.1% (4/33) of these TEN cases underwent steroid alone deceased. On the other hand, 57 patients with SJS and SJS-TEN received systemic steroids alone and only 1 (1.8%) died. There were 15 SJS and SJS-TEN patients who received combination treatment, and all survived. Mortality rate between using IVIG and steroid in combination or steroid alone had no statistical significance (Table 6).

## 4. Discussion

In this study, we enrolled a total 166 Han Chinese patients diagnosed with SJS, SJS-TEN overlap, and TEN from a tertiary medical center and Chinese literatures during 2006 to 2016. We evaluated underlying condition, causation, treatment, and clinical outcome. Mean age of SJS/TEN was 43.5 years, with little difference between SJS or SJS-TEN overlap and TEN. There was a male predominance in SJS or SJS-TEN overlap (male-to-female ratio 1.77 : 1) and TEN (male-to-female ratio 1.35 : 1). This observation was opposite to what Mohammed et al. found in Egypt and different from an earlier study which showed equally affected by male and female [42, 43].

There were 88.6% of SJS/TEN patients had drug relationship, and the major contribution was antibiotics, followed by anticonvulsants and allopurinol. The difference between the antibiotics and anticonvulsants was small. This result was similar to the comparison of Malaysia and Singapore in a review of Southeast Asia [41], only different in sequence of antibiotics and anticonvulsants, whereas Huang et al. found anticonvulsants as the most common drug which caused SJS/TEN in China, followed by allopurinol, antipyretics/analgesics, and cephalosporins [44]. Similarly, Li and Ma reported anticonvulsants and antibiotics to be the most common single drug in SJS and traditional Chinese medicines in TEN [45]. It is known that allopurinol, aromatic anticonvulsants, sulfonamide antibiotics, oxicam NSAIDs, and nevirapine have higher risk to induced SCARs [46]. Nevertheless, there were only some sulphonamides and none oxicam type of NSAIDs induced SJS/TEN in this study. This may due to prescribing habits of antibiotics in China and Taiwan, causing more penicillins and cephalosporins than the others [47–50]. Similarly, oxicam type of NSAIDs is less commonly seen in Chinese literatures of case series [48, 49]. Allopurinol was found to be a less common causality to induce SJS/TEN in this study, especially in northern China. From previous reports, *HLA-B*^∗^*58 : 01* was found positive in 93.3–100% of patients with allopurinol-induced SCARs whether in northern or southern China [51–54]. Moreover, the prevalence of carrying the risk *HLA-B*^∗^*58 : 01* allele was 0.0515–0.085 in China [55]. The discrepancy of the percentages between this study and literature needs further investigation. Chinese patent medicines were unique causative drugs to induce SJS/TEN in the Asian region [43, 56–59]. In our study, 5.4% of the SJS/TEN cases were related to Chinese patent medicine. Previously, Singapore was also reported to have more herbal medicine-induced SJS/TEN cases [41]. However, there are possibilities of adulteration with Western medicine in the component of Chinese patent medicine [60–62], which makes it hard to identify the exact causality and may cause bias. Patients also tend to received multiple drugs, including compound preparations of Western medicine or even antipyretic and analgetic in Chinese patent medicine [45]. Both of these would increase the possibility of adverse drug reaction and enhance difficulty of identifying offending drug.

In our study, 19% of patients did not have definite or possible relationship with drug according to ALDEN scoring system. The cause may be infection or idiopathic, and unfortunately there were no validation via further examinations. The annual incidence of SJS/TEN in the HIV-positive population is approximately 1000-fold higher than in the general population [63], and 4 patients with suspected causative drugs were HIV positive in our study. Infections are possible causations besides drugs. Reactivations of human herpesvirus 6 (HHV6) and cytomegalovirus were found in SJS/TEN [64, 65]. A case has been reported of a teenage boy diagnosed with SJS and primary Epstein-Barr virus infection without any attributing medication [66]. In addition, *Mycoplasma pneumoniae* infection may also be an additional cause of SJS. Watkins et al. and Olson et al. have reported *Mycoplasma pneumoniae* infection outbreak associated with SJS in children [67, 68]. Although there were some reports with malignancy-related SJS [69, 70], none of our non-drug-induced-SJS/TEN patients were found to have malignancy.

Withdrawal of offending drugs or treatment of causative infection, timely supportive treatment, immunomodulation, and management of complications and consequences are the most common suggested treatments [71]. In this study, all of the patients received systemic corticosteroid. Despite systemic corticosteroids remain a controversial treatment for SJS/TEN, it is the most commonly used medication across Asia [72–75].

Massive keratinocyte apoptosis induced by the intercellular death receptor Fas and Fas ligand is now considered to be the pathogenesis of SJS/TEN [76], yet IVIG inhibits keratinocyte apoptosis by inhibiting the FAS receptor [77]. IVIG was prescribed as an additional management in 45.8% of our patients, whether at the early or late stage of SJS/TEN, especially with much higher percentage in TEN (80.3%) compared to SJS or SJS-TEN overlap (19.7%). Apparently IVIG is a common option of treating SJS/TEN in China, especially in TEN for their extensive skin lesion involvement, and is usually in combination of systemic steroids instead of using alone. A score-based comparison study of clinical outcomes found that corticosteroid therapy combined with IVIG may lead to lower mortality when compared to corticosteroid alone [78]. However, several studies have shown limited success of IVIG in the clinical settings [79–82]. In our study, mortality rate in patients with TEN who received systemic steroids with IVIG comparing to those who received systemic steroids alone was 6.6% and 12.1%. However, this difference of mortality rate was not statistically significant. Application of intravenous immunoglobulins or systemic corticosteroids also did not improve the outcome of SJS and TEN in a study in Singapore [83]. Similarly, Lee et al. [84] demonstrated that the use of IVIG does not yield survival benefits in SJS/TEN overlap and TEN, even when corrected for IVIG dosages. Until now, the usage of IVIG in the treatment of SJS/TEN is still controversial. Recent studies have shown that immunosuppressive treatment with tumor necrosis factor-alpha (TNF-*α*) inhibitors may be helpful [85] and cyclosporin A is also safe and may contribute to rapid reepithelialization in patients with SJS/TEN [86–88]. The efficacy of using cyclosporin in treating SJS/TEN has recently validated with the decreased mortality rate both in adults and children [89–92].

There are several limitations in this study. First, we enrolled case reports only with careful checkup to prevent overlapping cases. However, ruling out the articles with case series also led to underestimation of SJS/TEN patients. Second, the mortality rate in our study is lower than international literatures which ranged from 10% to 70% [93, 94]. The possibility of lower mortality in this study may be due to underreported deceased cases of SJS/TEN from the Chinese literatures. In addition, the underlying severity of SJS/TEN in our study is unknown due to the lack of complete data of SCORTEN factors; hence, the efficacy of treatment needs to be further elucidated.

## 5. Conclusion

SJS/TEN is life-threatening drug adverse reaction, with higher prevalence rate in Asian than in Western populations in literature review. The most common offending drugs in our study are antibiotics, anticonvulsants, and allopurinol. IVIG in combination with systemic steroids is a common option especially for TEN in China. There was no significant difference in the mortality rate of TEN patients with or without IVIG adjuvant treatment.

## Figures and Tables

**Table 1:** Demographic data, treatment, and prognosis patients with SJS/TEN.

	SJS or SJS-TEN (*n* = 72)	SJS (*n* = 70)	SJS-TEN (*n* = 2)	TEN (*n* = 94)	Total (*n* = 166)	Odds ratio (95% CI)	*P* values
Age, y							
Mean ± SD	43.4 ± 21.7	43.5 ± 21.9	40.5 ± 11.5	43.6 ± 22.7	43.5 ± 22.3	—	0.967
Median (range)	48 (1–93)	48 (1–93)	40.5 (29–52)	44.5 (1–94)	45 (1–94)	—	—
Sex, *n* (%)							
Male	46 (63.9)	46 (65.7)	0 (0)	54 (57.4)	100 (60.2)	0.427 (0.406–1.435)	0.763
IVIG in combination, *n* (%)	15 (20.8)	14 (20.0)	1 (50)	61 (64.9)	76 (45.8)	7.024 (3.456–14.275)	<0.001
Pulse therapy	4 (5.6)	3 (4.3)	1 (50)	7 (7.4)	11 (6.6)	0.731 (0.206–2.600)	0.758
Death, *n* (%)	1 (1.4)	1 (1.4)	0 (0)	8 (8.5)	9 (5.4)	6.605 (0.807–54.071)	0.079

IVIG: intravenous immune globulin; SJS: Stevens-Johnson syndrome; SJS-TEN: SJS/TEN overlap; TEN: toxic epidermal necrolysis.

**Table 2:** Drug causality of SJS/TEN in China.

	SJS or SJS-TEN, *n* (%) (*n* = 72)	TEN, *n* (%) (*n* = 94)	Total, *n* (%) (*n* = 166)	Death, *n* (*n* = 9)
Culprit drug				
Allopurinol	**11 (15.3)**	**5 (5.3)**	**16 (9.6)**	**2**
Antibiotics	**12 (16.7)**	**37 (39.4)**	**49 (29.5)**	**5**
Penicillins^a^	2	10	12	1
Cephalosporins^b^	1	6	7	1
Carbapenems^c^	0	3	3	0
Quinolones^d^	3	3	6	0
Sulphonamides^e^	3	1	4	0
Others^f^	2	4	6	0
Unspecified^g^	1	2	3	1
Multiple drugs^h^	0	8	8	2
Anticonvulsants	**19 (26.4)**	**21 (22.3)**	**40 (24.1)**	**0**
Carbamazepine	12	17	29	0
Lamotrigine	4	3	7	0
Others^i^	3	1	4	0
Chinese patent medicines^j^	**6 (8.3)**	**3 (3.2)**	**9 (5.4)**	**0**
Industrial chemicals^k^	**0 (0)**	**3 (3.2)**	**3 (1.8)**	**0**
NSAIDs^l^	**3 (4.2)**	**5 (5.3)**	**8 (4.8)**	**0**
Multiple drugs^m^	**3 (4.2)**	**7 (7.4)**	**10 (6.0)**	**1**
Others^n^	**7 (9.7)**	**5 (5.3)**	**12 (7.2)**	**1**
Nondrugs^o^	11 (15.3)	8 (8.5)	19 (11.4)	0

NSAIDs: nonsteroidal anti-inflammatory drugs; SJS: Stevens-Johnson syndrome; SJS-TEN: SJS/TEN overlap; TEN: toxic epidermal necrolysis. ^a^Penicillins including amoxicillin (*n* = 5), amoxicillin with clavulanic acid (*n* = 1), ampicillin (*n* = 1), penicillin (*n* = 1), piperacillin (*n* = 1), and piperacillin-tazobactam (*n* = 3). ^b^Cephalosporins including cefalexin (*n* = 1), cefaclor (*n* = 1), cefuroxime (*n* = 2), cefoperazone sulbactam (*n* = 2), and cefotaxim (*n* = 1). ^c^Carbapenems including imipenem-cilastatin (*n* = 2) and meropenem (*n* = 1). ^d^Quinolones including ciprofloxacin (*n* = 1) and levofloxacin (*n* = 5). ^e^Sulphonamides including sulfasalazine (*n* = 2), sulfamethoxazole (*n* = 1), and compound of sulfonamides (*n* = 1). ^f^Others in antibiotics including azithromycin (*n* = 1), clarithromycin (*n* = 1), lincomycin (*n* = 2), doxycyclin (*n* = 1), and vancomycin (*n* = 1). ^g^Unspecified as not available, unspecified in contained group. ^h^Multiple drugs in antibiotics as concomitant use of multiple antibiotics. ^i^Others in anticonvulsants including oxcarbazepine (*n* = 2), compound of phenobarbital and scopolamine (*n* = 1), and phenytoin (*n* = 1). ^j^Chinese patent medicines including extract of *Andrographis paniculata* (*n* = 1), bupleurum granule (containing bupleurum, *Pinellia ternata* with ginger, radix scutellariae, *Codonopsis pilosula*, etc.) (*n* = 1), cough granule (containing loquat, opium poppy husk, stemona, mulberry bark, swallowwort rhizome, etc.) (*n* = 1), Gutong capsule (containing ginseng, resina draconis, scorpion, bungarus minimus, etc.) (*n* = 1), Honghua tablet (containing *Emilia sonchifolia*, *Hedyotis diffusa*, caulis spatholobi, etc.) (*n* = 1), sleeping capsule (containing lilium, *Acanthopanax senticosus*, caulis polygoni multiflori, Albizia julibrissin durazz, mother-of-pearl, etc.) (*n* = 1), and unspecified (*n* = 3). ^k^Industrial chemicals including acetochlor (*n* = 1), naphthalenedisulfonic acid dimethyl ester (*n* = 1), and trichloroethylene (*n* = 1). ^l^NSAIDs including analgin (*n* = 1), diclofenac sodium eye drops or tablets (*n* = 3), compound of paracetamol, aspirin and caffeine (*n* = 1), compound of paracetamol, aminophenazone, caffeine, and chlorphenamine maleate (*n* = 1), compound of paracetamol, aminopyrine, phenacetin, caffeine, and phenobarbital (*n* = 1), and ibuprofen (*n* = 1). ^m^Multiple drugs as different classification of drugs in concomitant use, including NSAID concomitant with antibiotic and anticonvulsant (*n* = 4), Chinese patent drug concomitant with antibiotic (*n* = 1), Chinese patent drug concomitant with unknown cold medicine (*n* = 2), and concomitant with multiple unknown cold medicine (*n* = 3). ^n^Others including calcium dobesilate (*n* = 1), methazolamide (*n* = 8), multiple antifungals (itraconazole and voriconazole) (*n* = 1), multiple antidepressant (amitriptyline and estazolam) (*n* = 1), and multiple antituberculosis drugs (*n* = 1). ^o^Nondrugs as absence of medication using history before onset.

**Table 3:** Comparison of the common drug causality between northern and southern China.

	Northern China, *n* (%) (*n* = 46)	Southern China, *n* (%) (*n* = 120)	Total, *n* (%) (*n* = 166)
Antibiotics	14 (30.4)	35 (29.2)	49 (29.5)
Penicillins	3 (6.5)	9 (7.5)	12 (7.2)
Cephalosporins	1 (2.2)	6 (5.0)	7 (4.2)
Quinolones	2 (4.3)	4 (3.3)	6 (3.6)
Others	8 (17.4)	16 (13.3)	24 (14.5)
Anticonvulsants	13 (28.3)	27 (22.5)	40 (24.1)
Carbamazepine	9 (19.6)	20 (16.7)	29 (17.5)
Lamotrigine	2 (4.3)	5 (4.2)	7 (4.2)
Others	2 (4.3)	2 (1.7)	4 (2.4)
Nondrug	1 (2.2)	18 (15.0)	19 (11.4)
Allopurinol	2 (4.3)	14 (11.7)	16 (9.6)
Multiple drugs	6 (13.0)	4 (3.3)	10 (6.0)
Herbal medication	2 (4.3)	7 (5.8)	9 (5.4)
NSAIDs	4 (8.7)	4 (3.3)	8 (4.8)
Others	4 (8.7)	11 (9.2)	15 (9.0)

NSAIDs: nonsteroidal anti-inflammatory drugs.

**Table 4:** The comparison of the common drug causality from cases in China with other populations in Southeast Asia^∗^.

Culprit drug	China (*n* = 166)	Malaysia (*n* = 162)	Singapore (*n* = 159)	Thailand (*n* = 60)	Philippines (*n* = 28)
Antibiotics	49 (29.5)	45 (27.8)	46 (28.9)	40 (66.7)	5 (17.9)
Penicillins	12 (7.2)	14 (8.6)	19 (11.9)	19 (31.7)	1 (3.6)
Sulfonamide	4 (2.4)	28 (17.3)	11 (6.9)	9 (15.0)	2 (7.1)
Others	33 (19.9)	3 (1.9)	16 (10.1)	12 (20.0)	2 (7.1)
Anticonvulsants	40 (24.1)	54 (33.3)	47 (29.6)	9 (15.0)	12 (42.9)
Carbamazepine	29 (17.5)	34 (21.0)	29 (18.2)	4 (6.7)	4 (14.3)
Lamotrigine	7 (4.2)	7 (4.3)	2 (1.3)	0 (0)	0 (0)
Phenytoin	1 (0.6)	13 (8.0)	14 (8.8)	4 (6.7)	5 (17.9)
Allopurinol	16 (9.6)	33 (20.4)	23 (14.5)	1 (1.7)	6 (21.4)
NSAIDs	8 (4.8)	10 (6.2)	14 (8.8)	4 (6.7)	3 (10.7)
Herbal medications	9 (5.4)	4 (2.5)	12 (7.5)	0 (0)	1 (3.6)

^∗^We compared the common drug causality from cases in China with other populations in Southeast Asia according to the previous literature report (41).

**Table 5:** Information of the deceased patients with SJS/TEN in this study (*n* = XXX).

Phenotype	Sex	Age, y	Underlying disease	SCORTEN	Culprit drugs	Treatment
SJS	M	51	Chronic renal failure, diabetes	4	Allopurinol	Systemic steroids
TEN	M	70	Nil	6	Antibiotics	Systemic steroids
TEN	F	58	Aneurysm, subarachnoid hemorrhage	NA	Antibiotics	Systemic steroids
TEN	F	67	Rheumatic heart disease, mitral insufficiency	NA	Antibiotics and compound with aminopyrine, phenacetin, caffeine, phenobarbital	Systemic steroids with IVIG use in the late stage
TEN	F	71	Coronary heart disease, hypertension, diabetes, diabetic nephropathy	4	Calcium dobesilate	Systemic steroids with IVIG use in the early stage
TEN	M	62	Hypertension, diabetes	NA	Antibiotics	Systemic steroids with IVIG use in the early stage
TEN	M	94	Coronary heart disease, cardiac insufficiency, hypertension, diabetes, interstitial lung disease	NA	Antibiotics	Systemic steroids with IVIG use in the early stage
TEN	M	62	Hypertension, diabetes, chronic renal failure, hyperuricemia	NA	Allopurinol	Systemic steroids
TEN	M	3	Nil	2	Antibiotics	Systemic steroids

IVIG use in the early stage ≤ 7 days of onset; IVIG use in the late stage ≥ 7 days of onset. NA: not available.

**Table 6:** A comparison of mortality rate between combination treatment of steroid with IVIG versus steroid alone.

Mortality	Steroids with IVIG (*n* = 76)	Steroids alone (*n* = 90)	Odds ratio (95% CI)	*P* values
TEN, *n* (%)	4/61 (6.6)	4/33 (12.1)	0.509 (0.119–2.183)	0.445
SJS and SJS/TEN, *n* (%)	0/15 (0.0)	1/57 (1.8)	—	1.000
Total cases, *n* (%)	4/76 (5.3)	5/90 (5.6)	0.944 (0.244–3.650)	1.000
