# Corrigendum to “The Epidemiology of Stevens-Johnson Syndrome and Toxic Epidermal Necrolysis in China”

**DOI:** 10.1155/2018/4154507

**Published:** 2018-06-28

**Authors:** Shang-Chen Yang, Sindy Hu, Sheng-Zheng Zhang, Jin-wen Huang, Jing Zhang, Chao Ji, Bo Cheng

**Affiliations:** ^1^Department of Dermatology, Xiamen Chang Gung Hospital, Xiamen, Fujian, China; ^2^Department of Dermatology, Chang Gung Memorial Hospital, Chang Gung University, Taoyuan, Taiwan; ^3^Department of Dermatology, The First Affiliated Hospital of Fujian Medical University, Fuzhou, Fujian, China

In the article titled “The Epidemiology of Stevens-Johnson Syndrome and Toxic Epidermal Necrolysis in China” [1], there was an error in the value of the deceased patients, in the legend of Table 5, where “(*n* = XXX)” should be corrected to “(*n* = 9).” The corrected table and its legend are shown below.

## Figures and Tables

**Table 5:** Information of the deceased patients with SJS/TEN in this study (*n* = 9).

Phenotype	Sex	Age, y	Underlying disease	SCORTEN	Culprit drugs	Treatment
SJS	M	51	Chronic renal failure, diabetes	4	Allopurinol	Systemic steroids
TEN	M	70	Nil	6	Antibiotics	Systemic steroids
TEN	F	58	Aneurysm, subarachnoid hemorrhage	NA	Antibiotics	Systemic steroids
TEN	F	67	Rheumatic heart disease, mitral insufficiency	NA	Antibiotics and compound with aminopyrine, phenacetin, caffeine, phenobarbital	Systemic steroids with IVIG use in the late stage
TEN	F	71	Coronary heart disease, hypertension, diabetes, diabetic nephropathy	4	Calcium dobesilate	Systemic steroids with IVIG use in the early stage
TEN	M	62	Hypertension, diabetes	NA	Antibiotics	Systemic steroids with IVIG use in the early stage
TEN	M	94	Coronary heart disease, cardiac insufficiency, hypertension, diabetes, interstitial lung disease	NA	Antibiotics	Systemic steroids with IVIG use in the early stage
TEN	M	62	Hypertension, diabetes, chronic renal failure, hyperuricemia	NA	Allopurinol	Systemic steroids
TEN	M	3	Nil	2	Antibiotics	Systemic steroids

IVIG use in the early stage ≤ 7 days of onset, IVIG use in the late stage ≥ 7 days of onset. NA: not available.
